# "The Hospital" Nursing Mirror

**Published:** 1897-09-11

**Authors:** 


### "The Hospital" Nursing Mirror.

BEING THE NURSING SECTION OF "THE HOSPITAL."

[Contributions for this Section of "THE HOSPITAL" should be addressed to the Editor, THE HOSPITAL, 28 & 29, Southampton Street, Strand, London, W.C., and should have the word "Nursing" plainly written in left-hand top corner of the envelope.]

### News from the Nursing World.

### THE QUEEN'S NURSES OF IRELAND.

THE Committee of the Jubilee Fund of the Queen's Nurses of Ireland are in the happy state of having accomplished a worthy object. The fund was opened by Lord Iveagh's gift of £12,500, and the committee fixed its hopes on raising that sum to £20,000. Subscriptions came in steadily, and a cheque from Mr. J. A. Scott of £86 from the Jubilee sports at Ball's Bridge brings up the amount to the coveted total. The beneficent reign of the district nurse will therefore soon be felt in many a lonely parish in our sister isle.

### THE SOCIETY FOR PREVENTION OF CRUELTY TO CHILDREN.

MR. EVELYN HUBBARD, M.P., is making a strong appeal for funds on behalf of this society. Not only has the recent inquiry cost the society something, but its supporters have withheld their subscriptions until the result was made known, to the great detriment of the society's income. A portion of the invested capital amounting to £5,000 has had to be realised in order to meet expenses, ordinary and extraordinary. The officials are jubilant over the verdict, and print in the *Child's Guardian* the compliments paid to the society by the press, but they pass over as "minor irregularities, chiefly personal," the grave recommendations of Lord Herschell's report. Nothing is said as to Miss Bolton's resignation, and her being replaced by a man with business training. Mr. Benjamin Waugh and Miss Bolton were both the recipients of valuable gifts presented to them by 200 enthusiasts a week or two ago, an occasion which afforded them an excellent opportunity of announcing their intention of following Lord Herschell's advice to the letter. That they have not done so will check the flow of contributions, and withhold from the society the countenance of many influential philanthropists.

### PAUPER NURSES OUT OF WORK.

IT is some years since that the world discovered that idle hands and mischief were near akin. Is it not strange, therefore, that amongst the many schemes for putting wrong right, the mischief-monger, idleness, should be systematically neglected? It is only recently that productive labour, with its healing influence on the moral nature, has been admitted into our prisons in place of the monotonous and useless treadmill and degrading oakum-picking, and healthy industry has still to invade that second stronghold of the "waster," the workhouse. With the advent of the new Local Government Board order on the 29th inst., the percentage of paupers who did something (very badly, it is true) for their living will be "out of work." Probably the paupers will not object to this; but however grievous wholesome discipline may be in the administration, there can be no two opinions as to its good effect, and regular work is an excellent disciplinarian. On the 29th a brighter day dawns for the physically sick and weak in the workhouse infirmaries; is it too much to hope that some day, not far distant, that a good time will come to paupers, who will then be taught to do such things as they can accomplish, and will in some measure reap the reward of their own industry. This has been done successfully in some French prisons, why not, therefore, in English workhouses?

### A STREET NUISANCE.

WOMEN are constantly sickened by the disgusting and disease-producing habit, so freely indulged in by men of the lower classes, of spitting in the streets. It was at all times horrible, but since the days that the microbe has been introduced to the public, it is intolerable. Americans, who were by all accounts worse offenders in this respect than our own countrymen, have acted very promptly in obtaining legal authority to put an end to it, and if a report that hails from San Francisco be true, they are applying their new powers rigorously. Mr. Bradbury, it is stated, a millionaire, has twice been convicted of expectorating in a tram-car. For the second offence he was sentenced to 24 hours' imprisonment without the option of a fine. When will a similar law be placed on our statute books?

### THE JUBILEE NURSES' HOME, MONTREAL GENERAL HOSPITAL.

LORD LISTER was chosen to open the new nurses' home now being built in connection with the Montreal General Hospital in honour of her Majesty's triumph year. The building is to be large, and nearly fireproof, and will accommodate 72 nurses. Long corridors run through the home on each storey, of which there are two, lighted by windows at either end. Galleries 10 ft. wide run across one end, and open off each floor, giving delightful opportunities to the inmates to indulge in the growing love for an outdoor existence. Single bed-rooms are provided, and there is a full complement of recreation and sitting rooms. The opening ceremony took place on September 2nd.

### PROPOSED NURSING HOME AT LICHFIELD.

THE rejoicings by which the Queen's long and glorious reign were celebrated at Lichfield were successful throughout. The fêtes were delightful, and the object of the permanent memorial popular. An enthusiastic spirit animated the committee, and the public subscribed liberally. At the final meeting of the General Committee a total of £1,006 was declared, of which sum £647 is now in the bank. The permanent memorial takes the form of a nursing home, with emergency beds. The Mayor had the pleasure of reading a letter from Mr. Seckham offering an excellent freehold site in Beacon Street. Archdeacon Scott said that £417 was already invested for the purpose of a nursing home, an executive committee was appointed, and the preliminaries were to be arranged as speedily as possible.

### NORTH WIMBLEDON NURSING ASSOCIATION.

The seventh annual report of the North Wimbledon Nursing Association shows a prosperous state of affairs. Subscriptions have come in to cover a small deficit of the previous year, and the support is more liberal and more widely spread than before. Many subscribers send a form to their bankers instructing them to pay their subscriptions when due. This is a great convenience, as the association employs no collector. A number of severe cases have been nursed with only six deaths—half the number of last year.

### LADY HENRY SOMERSET AND THE BRITISH WOMEN'S TEMPERANCE ASSOCIATION.

Some time ago Lady Henry Somerset placed the resignation of her post as president of the British Women's Temperance Association in the hands of the committee, as her opinion upon the Cantonment Act did not accord with that of her colleagues. She has withdrawn her resignation, as the committee unanimously consider the subject outside that for which the association was founded, viz., the promotion of temperance. The association is to be congratulated that she has done so. Lady Henry Somerset is an able, popular, and wealthy president, and many experiments for the reformation of drunkards are originated by her.

### NEW NURSES' HOME AT SHOREDITCH INFIRMARY.

The nurses of Shoreditch Infirmary will soon enjoy the well-situated, well-built, comfortable home that has just been erected. There is ample room in it for the whole staff, and careful provision has been made for study and recreation. The sitting-room is large, well-furnished, and carpeted, and has a piano and table for games, &c. The reading-room is immediately above on the first-floor; and off this floor is a sunny balcony. There are 54 separate bed-rooms neatly furnished. Each floor of the new home is connected with the main building by an iron gallery. The whole building is threaded with electric wires, and the cost has been £11,367.

### PRISONERS AND TRAINED NURSES.

The prisoners in Dublin are better off in one respect than are the inmates of London prisons, for when sick they are allowed the privilege of trained nurses obtained from the outside, and this rule applies both to male and female prisoners. Indeed, a story is current of the astonishment aroused in a male convict, who had not caught sight of a woman for many years, when he saw two trained uniformed nurses passing in at the gates to take charge of a sick prisoner. Turning to a fellow-convict he exclaimed with great emotion, "By St. Patrick, there's a woman!" and catching sight of the second following, he said, "and that's another!" At Grangetown Prison, the only female convict prison in Ireland until quite recently, there was a resident certificated nurse and midwife. But the Board is not sufficiently generous in the matter of salary to induce a certificated nurse to face the isolation of prison life. So that for the time being much valuable help is lost to the women prisoners. A midwife was called in a few days since to attend at the birth of a prison baby, and trained nurses are invariably requisitioned in case of any but slight illnesses. It would probably be just as economical in the end to appoint a permanent nurse and give her an adequate salary.

### THE GOUROCK NURSING SCHEME.

Those interested in introducing a trained nurse for the burgh of Gourock have shown true Scottish caution in their manner of setting to work about it. They have raised £118, and are disheartened by the apathy shown by those from whom they expected support. They have also been in correspondence with the promoters of the county scheme, the Greenock scheme, and the directors of the Greenock Medical Aid Society, and cannot see their way to associating themselves with any, so they have resolved to "gang their ain gait." And a very sensible gait it seems. They have some money; they will therefore engage a nurse for just so long as they can pay her, and when the purse fails the nurse goes and the experiment ends. If it fails no one will be any the poorer, and the money will have been spent on the object for which it was collected. If, on the other hand, a nurse be needed, a year's work will have taught the district her value, and most probably the necessary money will be subscribed to keep her, which is all that is required.

### NURSES' HOME FOR GARTLOCH ASYLUM.

The new nurses' home, which the Glasgow Lunacy Board intend to build in connection with their new asylum and hospital at Gartloch, is noteworthy as the first of the kind attached to an asylum in Scotland. It is proposed to spend between £5,000 and £6,000 on the construction, and a deputation from the House Committee will visit similar institutions in order that it shall be as well equipped, as convenient, and as handsome as possible.

### SHORT ITEMS.

Wishaw is to have a district nurse in honour of this year. Lord and Lady Belhaven take the initiative, and it is proposed to affiliate the new association with the Q.V.J.I.—The sanction of the Local Government Board has been asked for the following alterations in the nursing arrangements at Hampstead: A night sister is to be appointed at a salary of £35 to £38 per annum; a charge nurse, taking night duty and day duty alternatively every three months, at a salary of £27 to £30 per annum; and three assistant nurses at salaries of £18 to £20 each per annum.—Mrs. Grayson, matron of St. Peter's Workhouse, Ipswich, has been appointed superintendent nurse. She held a similar position before her marriage, and is a qualified midwife. Mrs. Grayson is relieved of her laundry duties in order to give her time for her new work.—The Steyning Infirmary Committee have made inquiries into the causes that led Miss Mowat, the head nurse, to tender her resignation. The result has been to show that Miss Mowat stands high in the estimation of her committee and her nurses, and that the unpleasant rumours of her unkindness towards her nursing staff, which made her unhappy in her work, are entirely unfounded. Miss Mowat has, therefore, withdrawn her resignation.—It is reported that the Yarmouth Guardians spend £200 a year in replacing nurses. A well-paid efficient staff would cost less money and be much cheaper in many ways, therefore people are asking why should the Guardians prefer to waste money instead of spending it to good purpose.

### Lectures to Surgical Nurses.

By H. A. LATIMER, M.D. (Dunelm), M.R.C.S. of Eng., L.S.A. of London, Consulting Surgeon, Swansea Hospital; President of the Swansea Medical Society; Lecturer and Examiner of the St. John Ambulance Association, &c.

### XIII.—DILIRIUM—DIFFERENCE BETWEEN HALLUCINATON AND ILLUSION—SURGICAL FEVERS: TRAUMATIC AND HECTIC FEVERS.

If these centres in the brain of which I was speaking in my last lecture are flushed with an extra amount, or are disturbed by a depraved quality, of blood, they become excited or depressed, and reveal their disturbances by outward evidences. When we are in health the will and intelligence stand in command of these centres, and impressions which reach us from without are sorted and arranged by them; but when we are ill, and especially when we are suffering from fever, not only are these impressions from without misunderstood by the brain centres, but actually the latter originate (just as they do in dreams) thoughts. The thoughts transfix themselves to the nerves flowing to the skin, eye, ear, tongue, and nose, currents of nerve force flow in the opposite direction to their natural course, and we feel, see, hear, taste, and smell things which do not really exist as facts, but which exist to us with all the sensations of reality. Looked at from this point of view, delirium is effective day dreaming. When you see patients on the operating table, being put under chloroform prior to their undergoing operations, you see the whole process of delirium originated and carried through to a speedy termination in sleep. You remember what often occurs there. The patients laugh, talk angrily, strike out and kick imaginary foes, and do other things of a like extraordinary nature. When you hear them talking and laughing, and see them doing these things, you have an example of delirium before you; they are holding interviews with imaginary people, who, to them, are real persons; and, according to their disturbed brains, they are acting rationally. For if one man is telling another a good joke he naturally laughs on hearing it, and "keeps up the fun;" and if he is being struck or being knocked about, it is a natural thing for him to retaliate and to strike and kick back. Another thing often happens when the mind is working wrongly; although it is receiving messages from the external world through one of the "five gateways," it misunderstands, twists, and disturbs these messages when it has received them. In this way the delirious person may think that a man who is really standing before him is a fiend or an angel, or that any common object which he is actually seeing is something of an extraordinary nature. Fix these two facts in your mind; that the affected person may see, hear, smell, and so on, with all apparent reality, things which do not exist at all, save in his imagination; and that seeing, hearing, smelling, and so on, things which really exist, his disordered brain may twist and distort these evidences of the senses and "make a fool of him." In the first case he is said to be suffering from a hallucination; in the second, from an illusion. If you understand these points fully you will find that they have a practical bearing when you are attending delirious people, and that they will furnish you with a clue to a line of treatment which is adopted when they are being nursed.

Inflammatory fevers in surgical cases have a tendency to assume definite types in such a way that we are enabled to classify them as distinctly as physicians are enabled to arrange medical fevers. Like the latter, they have their "breeding" times, or periods of incubation, by which is meant the time which elapses between the entrance of germs into the blood, and the manifestation of symptoms produced by the disturbance which they have effected there—but these periods are not of definite duration, and are not in any way constant. They arise by the processes called infection and contagion—much more frequently by the former than by the latter. When they are set up by infection the special germs, or other infective agents, have gained access to the blood, in most cases by being drawn into the air passages and lungs during the act of breathing. It is not at all difficult to realise how this should occur, for we all know how rapidly a person can be narcotised or poisoned by the fumes of chloroform, or of noxious gases which have been inhaled, and it is a reasonable thing to allow that the fine, and in many cases, undiscoverable atoms of infection, get into the blood by the same route. The air cells of the lungs, where the gases oxygen and carbonic acid are exchanged, are delicate membranes on which are spread millions of fine capillaries, and their very adaptability to the passage of these gases makes them quick absorbers of poisonous matters. When contagion comes into play the poison has been communicated by contact. Regarded from this point of view, disease following the bite of a snake or of a rabid animal would be of contagious origin as it could only be communicated directly by the absorption into the blood stream through a broken surface in the skin or the mucous membrane. You know you may be in the same room with a snake or mad dog and no harm will result unless you are bitten by either of them; but you run a risk of being infected if you associate with a person suffering from scarlet fever or measles.

It is customary to divide surgical fevers into classes according to their infectiousness or otherwise, and to call them septic or infective, in accordance to their communicative properties, or the reverse. The septic fevers owe their origin to a disorder of the blood set up by the absorption into it of poisons generated in wounds or diseased surfaces in the body, the word septic being derived from the Greek language, and meaning "having power to promote putrefaction." The poisons which cause them do not spread to other people by infection, and the effects produced in the system by their contamination of the blood are in proportion with the amount of them absorbed, so that if only a small amount has been absorbed there will only be a small amount of fever to follow. The simplest variety is known as traumatic fever, which shows itself by a rise of temperature some hours after the infliction of a wound or the performance of an operation. It is held that the slight rise of body heat which characterises this condition is due to an absorption into the blood of some material which has escaped from the blood at the injured part; and inasmuch as the temperature here does not rise high, and quickly falls again, reaching the normal point in one or two days, there is not much importance attached to it. Much more interest attaches itself to the consideration of the other two septic fevers—hectic fever and sapræmia—as they betoken a much more profound contamination of the blood, and are much more prolonged in their course. You will see a great many cases of hectic fever from one time to another, for it is the usual accompaniment of consumption, and of diseases where matter is being formed. The patient suffering from it does not feel very ill, and unskilful observers are apt to pass it over altogether, thinking that the flushed cheeks, the bright eyes, and the cheerful manners which often go hand in hand with it, are signs of health. But those affected with it complain of hot hands and feet in the late afternoon and evening, of feeling tired and restless in the early night, and of finding, on awakening in the morning, that they have been perspiring during their sleep. The systematic use of the thermometer throws a light upon these symptoms, for it shows that the temperature has risen to a feverish point in the evenings, and that it has fallen again in the mornings. The temperature does not rise to a high point, nor is the general disturbance of the system very great, because the fever is due to the continual absorption of only small doses of poison; were the doses larger we should have the graver condition of affairs which I am now about to speak of under the headings of septic traumatic fever and sapræmia.

### Post-Graduate Clinics for Nurses.

By a Trained Nurse.

### XXIX.—THE SERVING OF SICK DIET.

For many years now—after devoting ten years to dietetic studies and practical invalid cookery—I have been advocating hospital diet-kitchens, where nurses might devote three months of their training time in acquiring this needed branch. And I would have them begin with the lowest branch of saucepan cleaning, for no workmanlike work is ever done by persons who do not love their tools, and take infinite pride in their polish and perfection. No nurse would mind cleaning a saucepan were the process called sterilising; but, unfortunately, the old superstition that anything pertaining to saucepans must be derogatory still obtains, so we will suggest that in the diet-kitchen the nurses should be instructed in the sterilisation of stewpans.

I do not suggest for one moment that a nurse should include saucepan cleaning in her daily work. But I think she should learn how to do it thoroughly; for on this matter of saucepans much of the success of sick cookery will depend.

In the diet-kitchen scheme all the "extras" for patients which I propose for hospitals should be prepared, and then, indeed, the millennium of the ward convalescent would be accomplished. How often a sick person in a London hospital craves some "greens." It seems to be the special delicacy desired by the real Londoner. And how frequently, when the coveted permission is gained from the doctor, the greens come up so badly cooked that most of the portion is left on the plate. And the "little piece of chicken" so looked for is presented to the patient gravyless. A little delicately-prepared bread sauce and some nice gravy would transform the dish, and the benefit obtained from the meal would shorten convalescence. Another point is that badly-cooked food costs just as much as though it were well cooked. And no hospital can be said to be well nursed till the extra diets are prepared by the nursing staff. For nursing means "to nourish," and for this reason sick-cookery is essentially the province of the nurse.

It has been said that "genius is an infinite capacity for taking pains," and certainly it would be difficult to find a better definition of nursing than this axiom comprises.

In the preparation of an invalid's meal tray, I would have the nurse transform herself for the moment into the deftest, neatest parlourmaid. She would then look to it that only the daintiest napery were used on the patient's table. If the household boast lace betrimmed and frilled tray cloths, she would requisition them into the service of the sick. She would carefully see that the silver were at its shiniest height, and the glass polished to perfection point. Even if the patient take no wine, his tray should be adorned with a pink hock glass, just for beauty's sake. A small boutonniere attractively bestowed on his table, and his dishes trimmed with lace dish papers will prove well worth the few extra minutes devoted to this art of invalid table decoration.

Every new embellishment of the tray—each fresh polish added to the service—are so many attractive inducements to appetite. "Show me the table set forth by a nurse for her patient and I will tell you what manner of nurse she is," is the favourite formula of a woman who perhaps knows more of her art than any other living exponent. A nurse should herself adjust the tray for her patient and put the dainty finishing touches to it outside the patient's room. She should see that everything intended to be hot is served really hot, while things intended to be cold should fulfil their mission. How often in England one sees the butter-dish full of a half-melted oily, yellow, unattractive substance, when a small piece of ice would render it firm, cool, and delicious. In this matter of ice for hot weather the nurse should see that a supply is obtained twice daily. For there is no shadow of excuse, unless in far country wilds, for serving the sick with half-warm milk when this should be cold, or a drink of luke-warm unrefreshing water when the patient longs for a cool draught to relieve his thirsty throat. It is far better not to systematically supply a sick person with really ice-cold liquids. But all fluids intended to be cold should in hot weather be cooled with ice; for lukewarm beverages are not only unrefreshing, but they are sickly and nauseating. I always set down and "make a note" of that nurse who in summer weather serves grapes to her patient straight from the ice. What a contrast is such a delicious fruit to the inevitable grapes of the sick-room left for days by the bedside till they are positively hot and sickly. Now a few really cold strawberries, iced grapes, or a cold peach will not only relieve the dry, thirsty mouth, but act as a most desirable stomachic tonic, and help the patient's appetite. A nurse should be by nature a good "taster," able to detect shades of flavours. And she should invariably taste—outside the sick-room, and with her own spoon—the food she proposes to set before her patient. It is not a sign of good nursing when a patient has to say, "Nurse, I cannot take this broth, it is too salt," or "this tea tastes so strange, and it has too much sugar in it." In an over anxiety to keep up a patient's strength the nurse may easily give him too much, forgetting that it is by no means the quantity taken, but the amount assimilated, which is valuable to nutrition. For if the patient do not properly digest the food he takes he is wasting strength in the attempt. Modern requirements have made it the province of the nurse to keep watch and ward over her patient's food, so as to ensure the due digestion of that nourishment she has instructions to administer to him. Failing in his effort to make the best use—from an assimilative point of view—of his food, it becomes her duty to report the fact of its non-digestion to the physician, so that counsel may be taken as to substitutes. One of the golden rules of sick feeding is the axiom that no amount of trouble is too much to take toward ensuring the patient good and palatable food. Changes should be as frequent as possible, and small quantities given frequently will generally be the best plan, for the digestion is weakened by illness, and, added to this fact, lying in bed without the stimulus of exercise is enervating both to appetite and digestive capacity, so that the stomach is not able to manage large quantities at a time. I invariably find that nurses who have trained for a time in a children's or a maternity hospital have a knowledge of dietetics far superior to that possessed by those who have served their apprenticeship among adults. For the strict dieting of infants and children in hospitals is an admirable preparation for the reception by the nurse of the higher teaching of complex sick diet. And, after all, to speak generally, in most acute illnesses, the patient returns, so far as his diet is concerned, to his first childhood. In a children's ward the nurse becomes familiar with the cooking and preparation of the best patent and malted foods, and gains admirable practice in peptonising milk and other foods. Now, I have known many nurses who leave excellent adult training schools in London without even an elementary idea as to the peptonisation of a pint of milk, and who would have "to look it up in a book" were they told in private practice to prepare raw meat juice or meat pulp. That this is so is not so much the fault of the nurse as the defect in her training, which nowadays, despite our enlightenment, seems to leave out dietetics from nursing. To leave out sick-feeding from training is as the play with Hamlet gone from it.

### A Letter from Corea.

The country of Corea is one that in the past has been little known to the ordinary public mind. Recently it was brought more into notice as the cause of the war between China and Japan. It is not generally known that Corea was a cause of warfare to the Chinese a few centuries B C., and that that enterprising race carried on their campaign pretty much on the same lines as in 1894-5, unless perhaps the one doctor at Newchwang, provided solely with plaster for the possibly wounded out of 30,000 soldiers, may be regarded as an item of progress. Certain it is that 2,000 years ago the Chinese trusted more to banners and tom-toms and their power of inspiring awe through making faces and striking grotesque attitudes than to their swords, spears, and bows, and other legitimate weapons of antique warfare; just as in the last war they treated with disregard and a certain amount of contempt the more modern appliances for destruction, firing off their big guns at Yalu before the enemy's fleet were fully in sight, in hopes of deterring them from further advance by making a big noise. Their equally primitive ideas with regard to hygiene may be gathered from the following description of their houses. These are, with the exception of some of the better class, mean and uninteresting to the last degree, being simply mud huts, with a thatched roof of rice straw. Underneath are the fireplaces, which are lighted from the outside, and which extend the whole length and breadth of the floor. When the "hong," as they call it, has a fire in it, the floor of the hut is quite hot, and the whole interior, of course, very much like an oven. Moreover, the Coreans like a lot of company in a limited space, and bad tobacco, so that the atmosphere in which they live can hardly be imagined, especially when one remembers that ablutions are not considered by any means a necessary part of their daily toilet. Two by no means delicately-nurtured Englishmen once sought shelter in a rural Corean inn from an exceedingly heavy downpour of rain. They were shown to what looked like the door of an outhouse. The dimensions of this kennel were about 6 ft. by 4 ft. by 4 ft., there was a fire in the hong underneath it, and inside of it were already four natives, squatting on their haunches and smoking; then there was no window and no ventilation to speak of. The rain was certainly bad, but it was much preferable to the shelter offered. The house of a Corean gentleman, if there be such a person, is quite habitable, at any rate under English management. The Anglican mission at Seoul under Bishop Corfe occupy a very nice one. It has a tiled roof, fine rafters inside, and a wooden floor raised about three feet from the ground. In the same compound are the servants' apartments, separate from the main building, and now used as a hospital and a printing house. There is also a good garden, and adjoining this are the house and grounds belonging to, and occupied by, the English surgeon, E. H. Baldock, Esq., late of Guy's Hospital. The hospital gets a tremendous amount of work in the year, and both doctor and European nurses have their hands full. There is always some difficulty with the patients when they first come into the hospital, owing to their objection to sleep in a bed; they always sleep on the floor in their own huts. It is only the male patients who are admitted to the hospital in the mission compound; there is, in deference to the native customs with regard to women, another hospital for women near the English Consulate and church. This is presided over by a lady doctor, and has a staff of English nurses. In 1894 or 1895 a very nice ward was given to the women's hospital by Mrs. Bishop, the well-known writer and traveller.

The state of some of the side streets of Seoul is enough to make the hair of any ordinary sanitary authority stand permanently on end. The best pavement that any street can boast is mud hardened by the innumerable patter of feet, naked in summer, but protected by the native peculiar footgear in winter. Of course in summer it is dry, and in the winter it is frozen, and as there is no wheel traffic the mud pavement answers very fairly for most days of the year; the great drawback is the fact that at least one-third of the very narrow gangway is occupied by a drain, not a high class swift-running drain, with pipes and tiles, but a sluggish, shallow, open, mud drain, into which are thrown all sorts of refuse and dirt too dreadful for words. It is a wonder there is no regular epidemic, but the drains being open, though exceedingly objectionable, are less unhealthy than if they were closed. Generally, the drain runs right down the middle of the pathway, so that travelling requires a certain amount of caution, and in damp weather or in the dark it is absolutely dangerous.

### Presentations.

Mile End Infirmary.—On Tuesday last, at the Mile End Infirmary, a very interesting ceremony took place in the presentation to Miss Stewart, assistant matron, of a massive marble clock, bearing the engraved inscription—"Presented to Miss L. Stewart by the Nursing Staff of the Mile End Infirmary upon her retirement, as a mark of respect and esteem"—and a pretty flower vase by the nursing staff of the infirmary, on her leaving to take up the important position of Matron of the City of London Infirmary. The presentation took place in the spacious nurses' recreation-room, where all the nurses were assembled. Mr. T. A. Spratley (steward of the infirmary), in making the presentation on behalf of the whole of the nursing staff, said it was with mingled feelings of regret and pleasure that they had met there to bid farewell to their able leader and tutor. Whilst regretting the severance, they rejoiced at the cause, which was to fulfil a higher and more responsible position in a neighbouring institution, where they had no doubt Miss Stewart would soon win the esteem and respect of those under her. Miss Stewart, in replying, said she valued much the kind words spoken on behalf of the nurses and the handsome timepiece they had given her, more for the kind feelings which prompted it than for its intrinsic worth, and hoped that the nurses would continue to carry out their duties in a conscientious and kindly manner at all times.

### Minor Appointments.

Cuddington, Surrey.—The following Charge Nurses were appointed at the Joint Isolation Hospital on September 6th, 1897: Miss Mary Brooks, who was trained at Durham County Hospital, and was subsequently assistant nurse at the same hospital; she has since been nurse at Bromley and Beckenham Joint Hospital and at the South Metropolitan District School Infirmary, Herne Bay. Miss Agnes McIntosh, who was trained at Holborn Union Infirmary, and was afterwards assistant nurse at North-Western Hospital and nurse at Cheam Hospital, Surrey.

South-Eastern Fever Hospital, E.C —On September 1st the following Charge Nurses were appointed at the above institution: Miss Janetta Matthewson, trained at the Royal Infirmary, Dundee; and subsequently sister at Blackburn and East Lancashire Infirmary; Miss Dora Scott, trained at Bradford Royal Infirmary; and Miss Mary C. Bishop, trained at St. George's Hospital, S.W.

Fulham Union Infirmary.—On September 2nd Miss Alice Violet Stewart was appointed Charge Nurse at this infirmary. She was trained for three years at the Devon and Exeter Hospital, and subsequently held temporary appointments as Sister of children's ward, York and County Hospital, and staff nurse Royal Infirmary, Derby.

North Wimbledon.—Miss M. C. Henderson was appointed District Nurse to North Wimbledon on July 12th. Miss Henderson is a member of the Royal British Nursing Association, and is on the Royal National Pension Fund.

Truro.—On September 2nd Miss F. J. Mitchell was appointed Charge Nurse at the Royal Cornwall Infirmary. She was trained at the Leeds General Infirmary.

### A Book and its Story.

### THE ROMANCE OF ISABEL, LADY BURTON.

Mr. Wilkins has chosen a most appropriate title* for his work. The life of Isabel, Lady Burton, was a romance; and the manner in which the author of these volumes has treated it, leaves, in our opinion, nothing to be desired, and places Lady Burton's biographer in the foremost rank of writers of the day.

Without entering upon any of the controversial questions into which Mr. Wilkins has unavoidably been led, we shall, without further preface, call our readers' attention to what strikes us as the salient points of this attractive narrative. The characters of Lady Burton and her husband have, during the last few years, been bitterly attacked and keenly defended. Burton was originally intended for the Church, and went to Oxford with the view of taking holy orders; but his previous education, the "free Bohemian society" in which he had been brought up, had entirely unfitted him for the Church. Expelled from Oxford, he was not long in finding a more suitab'e career in India, where, having obtained a commission in the Bombay Native Infantry, he remained for several years, deeply engaged in the study of Oriental languages and customs. In 1850 he returned to Europe, and it was during a visit to Boulogne that he first met Isabel Arundell. The future Lady Burton, who was then a girl of 19, was there at that time with her parents, Mr. and Mrs. Henry Arundell. She had already given indications of a strangely complicated nature, one which combined the characteristics of the commonplace society young lady of the first portion of this century with a dash of the Bohemianism which is fashionable at the present time. Thus she had gone through a season in town, rejoicing in her first ball at Almack's, where she appeared in "white tarlatan over white silk, skirt looped up to my knee with a blush rose." And so on. But she and her sisters did "wild things." Moreover, she had an ideal lover of whom she was always thinking, an imaginary being whose coming had been foretold by a gipsy. At last, one day at Boulogne, the ideal appeared in the shape of Richard Burton, the man "with the brow of a god and the jaw of a devil," tall, broad, thin, muscular, with a weather-beaten face. "He looked at me as though he read me through and through. I was completely magnetised." It is here that the genuine romance of Isabel Lady Burton begins. They were soon introduced; but for four years after leaving Boulogne they saw nothing more of each other. Isabel mixed in London society, had admirers, and received many offers of marriage. But she was waiting for Burton, on whom she had set her heart in the most romantic fashion. At last he returned to England; he returned poorer, and dispirited by official rows and every species of annoyance; "but he was still—had he been ever so unsuccessful, and had every man's hand against him—my earthly god and king, and I could have knelt at his feet and worshipped him." Burton was under a cloud, the victim of calumny and suspicion, with all the world looking askance at him; but, instead of turning from him, Isabel Arundell seized that very moment for asking her parents' leave to join her fortunes to his. "Then it was," says Mr. Wilkins, "that Burton proved the strength of a woman's love." Her mother would not hear of such a marriage; and for about a year Burton pressed her to make a runaway match of it. Burton, however, suddenly left her, without warning or farewell, merely leaving a short note, in which he said that she must choose between her mother and him, that he was going to Salt Lake City, and that if, on his return, she had not made up her mind, he would go to India and return no more. "This was the last straw to Isabel, and for a time she broke down utterly." But at Christmas, 1860, when on a visit in the country, she saw in the *Times* an announcement that Burton had arrived from America. She found an excuse for going to London, where he met her, and where they decided that in three weeks, with or without leave, the marriage should take place. During these three weeks she drew up a series of rules for her future life which are so characteristic that some notice must be taken of them. "I have undertaken a very peculiar man," she says, and then goes on to arrange how she can make their life together happy. She is to be the "companion, friend, and adviser, and confidant." Their home is to be "snug." If it be ever so small and poor, there can always be a certain *chic* about it. "Attend much to his creature comforts; allow smoking or anything else; for if you do not somebody else will." She knew Burton's character thoroughly when she penned the last sentence of her "rules," in these words, "nothing would weary him like stagnation." As they were poor they were to do "any amount of study and publishing," and "when successful we will travel."

The marriage took place on Tuesday, January 22nd, 1861. It was followed by a merry little breakfast at the house of Dr. Bird. Miss Bird has described to Mr. Wilkins how, after the breakfast, the newly-wedded pair walked off to St. James's, and settled down without any fuss. In Lady Burton's own words, "We went to Richard's bachelor lodgings, where he had a bed-room, dressing-room, and sitting-room; and we had very few pounds to bless ourselves with, but we were as happy as it is given to any mortals out of heaven to be."

Some years of wandering and frequent separations followed the marriage, during which we find Mrs. Burton writing, in September, 1866, when there had been some idea of her coming home, "I have just domesticated and tamed Richard a little, and it would not do to give him an excuse for becoming a wandering vagabond again. They were then in Brazil. In 1869 Burton was appointed Consul at Damascus, mainly through the exertions of his wife; and there the pair lived a most unconventional life. But Burton's position at Damascus gradually became untenable. After a series of misunderstandings with the Foreign Office, and of errors, there can be no doubt, on the part of the Consul, Burton was suddenly recalled. Burton was a man of genius, but as Consul at Damascus he had become impossible. He left for England at once, and his wife followed him. Just ten months after his recall from Damascus he was appointed Consul at Trieste, an office far below his abilities, but which, steeped in poverty as he was, it would have been folly to refuse. After this the story becomes one of failing health on the part of both, journeys in search of cures, and, with mutual love and confidence undiminished. nay, growing even stronger, the gradual nearing of the end, It was at this sad time that the translation of the "Arabian Nights" was begun, Burton working hard, helped by his wife, who assisted him in compiling the index, and, what was a still more arduous task, looking after the financial part of the undertaking. Soon after this Burton received his knighthood. After this the Burtons reconciled themselves to remain at Trieste. The end came on October 20th, 1890, when Burton died suddenly, in the circumstances which have been described in the "Life."

A few more years, and in March, 1895, Lady Burton followed him into the darkness beyond, still thinking to the close of the loved one who had gone before. The romance of Isabel Lady Burton was at an end. "A smile of peace and trusting came over her face, and with a faint sigh she breathed her last. She had heard the 'tinkling of his camel's bell.'"

* "The Romance of Isabel, Lady Burton." Told in part by herself and in part by W. H. Wilkins. (London: Hutchinson and Co. 1897.)

### On Wardmaids.

The wardmaid is a very important and a very independent person. She reveals the latter proclivity when she chooses hospital work rather than the more cultured surroundings of ordinary domestic service. She knows that in an institution she will have regular times off duty, and that no whims of the missus will ever snatch away her cherished "evening out," which she would have you believe is a right accorded to her by Act of Parliament. Her work is regular and defined, and anything beyond its ordinary routine at once constitutes a grievance for which she can demand redress. She purchases her freedom by the comparative lowness of her wages; but this enters into her calculations, and she ceases to aspire beyond the limits of her £14, and settles down to the full enjoyment of her other privileges.

The wardmaid is generally young, but occasionally attains to middle age. When such is the case you might as well entreat the stars to alter their courses in the heavenly way as beg the personage who has dominated a ward for twelve years or more to rearrange the order of her work. The new sister, who usually feels that the ward she has come to reign over requires radical and immediate alterations in all its departments, will be unwise to try her novice hand in reorganising the antique system by which an old wardmaid performs her daily round of duty. The hot reformer must console herself with the thought that it is just possible old brooms sweep clean sometimes, and try to believe there may be hidden advantages lurking under the apparent lack of method which to her new-born notions is as salt rubbed on a sore place.

The wardmaid being a person of fixed principles it is satisfactory to find she spares no trouble to uphold them. She will carefully sweep round a little tuft of hair because she knows the patients should not throw their combings on the floor; she will allow a splash of milk to dry upon the kitchen table because a probationer has spilt it; and when the beef-tea is allowed to boil over (greatest of all crimes) it will require a firm tone of authority to convince her that it is not the culprit's due to be ordered to blacklead the grate and whiten the hearth. The hand that scrubs the kitchen rules the ward—or tries to do so.

It is worth observing that no wardmaid entirely approves of the chosen time for coming down in the morning, it would always suit her better if it were ten minutes later, and she thinks it well to frequently enforce her views upon the subject in a practical manner, whilst on Sunday it becomes one of her fixed principles to be systematically late. It is equally difficult to obtain her approval of the hour specified for going to bed. She is, moreover, very particular about her food and has a special aversion to mince, preferring to starve rather than taste this harmless article of diet. She never hesitates to show her disapprobation, making full use of the British privilege of grumbling, and always expressing her candid opinion in audible undertones. Frequently she is endowed by nature with the power of mimicry, and she seeks to cultivate the gift whenever an opportunity occurs.

This simple Cockney lassie may constantly be seen leaning on her elbows on terrace, balcony, roof, window-sill, or other coign of vantage her hospital affords, admiring the view, studying human nature, and planning her next new hat, for our wardmaid is nothing if not smart. Her evening off is signalled by the appearance of a fringe in chrysalis form lying obscurely in the folds of her cap, preparing to burst forth a few hours later in full glory, surmounted by a large black hat with nodding plume or fantastic flower. And the uninitiated wonder how it is done on £14 per annum.

The wardmaid is a type of a democratic Tory. She looks with eye askance upon all innovations, and acknowledges only supreme authority, holding lesser lights in profound contempt. She craves to be a law unto herself, and would live up to her own code honourably and zealously. Not having yet arrived at this condition of self-government, the difficulty consists in framing laws by which these strange, unruly mortals can be kept disciplined and in order. The question is as difficult of solution as the demand for an eight hours' day, for we have to contend with the spirit of the age, and are forced to face problems which are puzzling all employers of labour. At present there seems no deliverance, and we still wait for the halcyon days when master and man, matron and maid, will be of one mind and one spirit.

### Everybody's Opinion.

[Correspondence on all subjects is invited, but we cannot in any way be responsible for the opinions expressed by our correspondents. No communication can be entertained if the name and address of the correspondent is not given, or unless one side of the paper only is written on.]

### PROBATIONERS AND NURSES UNDER THE NEW ORDER OF THE LOCAL GOVERNMENT BOARD.

"A Watcher" writes: By the recent action of the Local Government Board a number of superior positions are thrown open to nurses who excel in their profession. This will doubtless not only improve the status of the workhouse infirmary nurses, but will attract a larger number of young women to their ranks. Probably, the new candidates will be of a superior class. There is a great pitfall before the feet of the unwary probationer, as was pointed out at the end of Article III. in the "Nursing of the Sick in Workhouses" ("Mirror," August 21st). It is clear from the general order issued by the Local Government Board that anyone who is in future engaged as a nurse must have "had such practical experience in nursing as may render him or her a fit and proper person to hold such office," and we do not yet know what experience will be accepted as sufficient under this clause. If "training" is required it is most important that a probationer bofore signing an agreement should make sure that she will be provided with such training as will be accepted as a qualification. But nurses should still further bear in mind that however skilful they may become they will not in the future be allowed to attain the dignity of superintendent nurse unless they have been trained "in the medical and surgical wards of any hospital or infirmary being a training school for nurses and maintaining a resident physician or house surgeon." It is, then, most important at the very beginning of their career that those who aspire to the higher posts should not waste their time as probationers in the smaller infirmaries. Future promotion depends, then, on the choice a probationer makes at the commencement of her career of her training school, and not on any personal merit or ability or willingness to learn. A great hardship is thus inflicted on those who have already become probationers at infirmaries and hospitals not qualified to give the needed instruction, and any suggestions how to ameliorate it would be helpful.

### Where to Go.

Midwives' Institute and Trained Nurses' Club, 12, Buckingham Street, Strand, W.C.—On Monday, September 13th, at three o'clock, Mrs. Hewer will lecture on "The Care of the Infant," admission 2s. Midwifery lectures on Mondays and Thursdays at four o'clock. Admission 2s. 6d.

### For Reading to the Sick.

"THE FOUNTAIN HEAD."

**Verses.**

LOOKING unto Jesus,
Wonderingly we trace
Heights of power and glory,
Depths of love and grace.
Vistas far unfolding
Ever stretch before,
As we gaze, beholding
Ever more and more.

Looking up to Jesus
On the Emerald Throne!
Faith shall pierce the heavens
Where our King is gone.
Lord, on Thee depending,
Now, continually,
Heart and mind ascending,
Let us dwell with Thee.
—*Frances R. Havergal.*

Satisfied with Thee, Lord Jesus,
Is my heart;
And I'm longing to be with Thee,
Ne'er to part,
Sharing with Thyself Thy glory
Where thou art. —*L. W.*

As from strength to strength I go
Through Baca's vale,
Thy streams of life and grace, I know,
Will never fail.
Lord, thou has filled my pools with rain
From God's deep well;
And of its living spring I fain
Would ever tell.
Oh, take me to the fountain-head
On yonder shore,
That I may praise Thee there instead
For evermore.

**Reading.**

Moderation in the use of power is a sign of true greatness of soul. There is a calm reservation of strength about genuine excellence which makes its possessor patient towards the weakest and ready to encourage the good that is in the worst. Never is a man so Godlike as when he thus acts. This is an aspect of the Divine character we are slow to appreciate, and yet we see it in Nature, Providence, and Grace. So far as we succeed in grasping the thought we feel a mighty spell controlling our wills, fascinating our inmost being, and drawing us upwards by bands of love with the cords of a man. How exquisitely is it seen in the material world! When the furious hurricane sweeps all before it in its irresistible course, or when the lurid lightnings flash and deep thunders roll, or if the ground heave beneath us with convulsive throes of an earthquake, or the pestilence stalks abroad, slaying thousands with its malarious breath, the most sceptical feel themselves in the presence of a Power greater than their own, and the stoutest hearts quail before the display of forces in Nature, which Nature's God alone can control. Still, sights and sounds like this often leave us no better than they found us. It is Nature in her wilder forms that woos and charms and elevates the spirit. The view of the ocean rolling with pride and bursting impotently upon the iron-bound coast thrills the most thoughtless with awe and wonder; but when we find its mighty energies regulated by the gentle moon's attraction, and held in check by the law of gravity, we form a far nobler conception of the wisdom and power of Him who has fixed the bounds of the sea by a perpetual decree that it cannot pass.—*Rev. W. Burnett.*

### Notes and Queries.

**The contents of the Editor's Letter-box have now reached such unwieldy proportions that it has become necessary to establish a hard and fast rule regarding Answers to Correspondents. In future, all questions requiring replies will continue to be answered in this column without any fee. If an answer is required by letter, a fee of half-a-crown must be enclosed with the note containing the enquiry. We are always pleased to help our numerous correspondents to the fullest extent, and we can trust them to sympathise in the overwhelming amount of writing which makes the new rules a necessity. Every communication must be accompanied by the writer's name and address, otherwise it will receive no attention.**

*Assistant Nurses.*

(375) Can you tell me of any general hospital, not infirmary, in London or near, where assistant nurses are taken?—*H. C.*

See our advertisement columns, and advertise yourself.

*A Fair Chance to Probationers.*

(376) Having read the article on "The Nursing of the Sick in Workhouses," may I ask if those probationers who have just entered on a three years' agreement, such as we have in this infirmary at present, are not to have the chance of becoming superintendent nurses because the matron and doctors in charge do not hold classes and give lectures? (2) Should not every matron where there are two or more probationers be compelled to give her nurses as fair a chance as possible?—*A Nurse.*

1. To be qualified for the appointment of superintendent nurse, a person must have "undergone for three years at least a course of instruction in the medical and surgical wards of any hospital or infirmary, being a training school for nurses, and maintaining a resident physician or house surgeon." Three years' "instruction" is necessary, consequently those probationers above referred to cannot become superintendent nurses. (2) There is no authority to compel matrons to hold classes and give lectures. Probationers should see to this before signing agreements.

*Is Midwifery an Essential Qualification?*

(377) Could any readers of THE HOSPITAL who have had experience in district nursing tell me whether midwifery is an essential qualification for obtaining good district appointments, or if well-trained general nurses with district experience would stand a fair chance of getting good first appointments?—*D. M.*

The midwife's diploma is always valuable, but not absolutely essential.

*Experience in Paris.*

(378) 1. Having had three years' training, we are anxious to get some experience in Paris. Would you kindly give us your advice, (2) and the addresses of hospitals or nursing homes in Paris where we could get the best experience?—*Two Nurses.*

1. The Lady Superintendent of Nurses (Miss Newman), Hertford British Hospital, Rue de Villiers, Levallois-Perret, Paris, might be able to advise you; and the Secretary, Y.W.C.A., 88, Faubourg St. Honoré, Paris, would give reliable information about board and lodging. Be careful to have work promised before you leave England, and remember that young women must be most circumspect in their behaviour abroad, as there are many pitfalls laid for unwary feet.

*Probationer in a Children's Hospital.*

(379) 1. Would you kindly tell me if there is a good children's hospital in Liverpool or district? 2. Do you think I should have much difficulty in getting into a hospital of that kind?—*L. S.*

1. The Liverpool Infirmary for Children, Myrtle Street, has 80 beds. Write to the Lady Superintendent for particulars. 2. Very little, if suitable for the work.

*Holiday Home for Epileptics.*

(380) Will you kindly tell me the name and address, and if possible the terms of a convalescent home where a young man, subject to epileptic fits, might stay for a holiday?—*B. Nevill.*

Write to the secretary of the National Hospital for the Paralysed and Epileptic, Queen's Square, Bloomsbury, W.C. This hospital has recently opened a charming convalescent home at East Finchley for its own patients. As a certain number of wards are reserved for patients who pay 21s. weekly, probably the young man would be received as such for the home.

*Hospitals and Infirmaries.*

(381) Would you kindly tell me the difference between a hospital and an infirmary?—*G.M.K.*

The words "Hospital" and "Infirmary" are interchangeable terms, the meaning varying according to locality.

*Addresses of Firms.*

(382) I wrote last Monday to three different firms which advertise in the "Mirror"—Vimbos Co., Riddle and Co., King's Cooked Oatmeal—asking them for a sample on receipt of postcard (as they say they will do), but have received no answer from any of them, so write to ask if they are good firms. (2) Please give address of Evans, Gadd, and Co. (3) Could you recommend anything to prevent hair falling out?—*Sister Gertrude.*

(1) They are good firms, and will probably send samples on as soon as they can. (2) Messrs. Evans, Gadd, and Co., 97—100, Fore Street, E.C. (3 If the hair falls badly it denotes something wrong with the general health, and medical advice ought to be sought. Unless the hair is actually diseased, a stimulating and cleansing lotion applied to the roots weekly will keep the hair in good condition. There are many washes to be bought, and few are better than the old-fashioned borax, camphor, and rosemary, which has the double merit of being cheap and easily prepared at home.

*The Mary Wardell Convalescent Home.*

This institution is in Stanmore, Middlesex, not in Essex, as stated in last week's "Notes and Queries."

